# The Management of Hemorrhage after Tonsil Operations

**Published:** 1895-05

**Authors:** A. W. Calhoun

**Affiliations:** Atlanta, Ga.; Prof. Diseases of the Eye, Ear, Throat and Nose, Atlanta Medical College


### THE MANAGEMENT OF HEMORRHAGE AFTER TONSIL OPERATIONS.

By A. W. CALHOUN, M. D., LL. D.
Prof. Diseases of the Eye, Ear, Throat and Nose, Atlanta Medical College.

The frequency of alarmingly profuse hemorrhage after tonsil operations, with an occasional fatal result, makes this a subject of exceeding interest to the surgeon. Such accidents should not, however, stand as obstacles in the way of performing these operations, for the necessity for operating, is both frequent and urgent. Especially in children is the enlarged or hypertrophied tonsil very often met with, and the evil effects of the disease are so apparent to the medical and even non-medical observer, that it often calls for prompt action on the part of the physician. I do not intend here to enter into a discussion of the disease itself, but I would say, that within the range of surgical diseases, I do not know of any affection that so often produces a train of more distressing symptoms than a typical case of enlarged tonsils; nor do I know of any operative procedure that gives more decided or permanent relief than their proper removal.

The ordinary hemorrhage following the operation is of but small consequence, as with a little patience and care, the blood soon ceases to flow of its own accord, or, if need be, a gargle of cold salt water suffices to arrest it in the course of a few minutes. But when there flows from the cut surfaces a steady stream of blood, continuing for hours, and when the frightened patient becomes each moment weaker and more nervous and more difficult to manage, then it is, that the tact and nerve and skill of the surgeon display themselves to the greatest advantage. A typical case of violent hemorrhage from an operated tonsil, uncontrolled by the ordinary remedies and means, is an experience that no doctor will willingly confront the second time. Fortunately these very severe hemorrhages rarely occur, but they *do* occur, and the operator should be prepared to contend with them at any time. The operation is chiefly necessary in children from 3 or 4 to 10 or 12 years of age, and with an experience of over 3,000 tonsil operations, I have seen but few cases of alarming hemorrhage in children.

The inference, therefore, is that young children are not very liable to dangerous hemorrhage—which, for obvious reasons, is a fortunate exemption. But it is in the adult that the great danger lies, because of the hardened tissues of the gland and the increased number and size of the blood vessels over that of the child.

Generally, the tendency to serious hemorrhage manifests itself immediately after the operation, or at latest, after a few hours. In one of my cases, however, secondary hemorrhage occurred five days after the operation.

There can be no objection to the trial of the various styptics, for they do in some instances arrest the flow, but they too often fail and, even when successful, they leave behind most unpleasant results.

The actual cautery applied directly to the bleeding surface has been recommended, but the objections to its use are so obvious that I could not suggest it. But I can recommend compression as a remedy. Pressure directly applied to the wound is the most satisfactory of all means. Pass the forefinger, the end of which is covered with a piece of moistened sponge, or absorbent cotton, into the mouth and carefully cover the cut surface, and with the wound between the forefinger of the one hand and the palm of the other hand placed externally, exercise a gentle but steady pressure. The hemorrhage ceases immediately, but recurs upon the removal of the pressure. It is now a matter of courage and confidence on the part of the patient, and of physical endurance on the part of the doctor. In most cases, pressure continued for ten minutes to one or two hours suffices to permanently arrest the hemorrhage, but in rare instances it must be continued through 12 to 24 hours. In these last cases assistance must be called in, so that the persons exercising the pressure can be rested at intervals of half an hour. But I have never known this mode of checking the hemorrhage to fail and I can confidently recommend it to any one having such a case.

I read a suggestion in some medical journal not long since, which I am inclined to think has some merit in it, though I have given it no trial; that is, the hypodermic injection of small doses of apomorphia, with the view of inducing nausea, which is supposed to exercise a beneficial effect in lessening the hemorrhage. It is worthy of a trial, for I know by experience that extreme nausea bordering on fainting, or actual syncope itself, will arrest the hemorrhage. Before learning to rely so implicitly upon pressure, I recall several cases, in which the nausea, on account of the loss of blood, fright and nervous shock, became so intense as to be in itself alarming. In three of these cases, after unsuccessfully trying every remedy at my command and when death seemed imminent, syncope followed (in one while lying in bed), and instantly the hemorrhage ceased and did not recur upon reviving the patients.

But since I have learned what an infallible remedy the pressure is, I have had no such bad cases as just mentioned. If the pressure is properly done and continued long enough, a fatal result can not ensue.

